# Youth Who Have Lived in Alternative Care in Nigeria, Zambia, and Zimbabwe: Mental Health and Violence Outcomes in Nationally Representative Data

**DOI:** 10.1016/j.jaacop.2023.05.002

**Published:** 2023-06-06

**Authors:** Sarah Elizabeth Neville, Oladoyin Okunoren, Thomas M. Crea

**Affiliations:** a Brown University School of Public Health, Providence, Rhode Island; b Boston College School of Social Work, Newton, Massachusetts

**Keywords:** foster care, residential care, orphanage, kinship care, child protection

## Abstract

**Objective:**

We explore whether having previously lived in alternative care (foster, kinship, and/or residential care) is linked to sexual risk taking, mental health, and experiencing violence in Nigerian, Zambian, and Zimbabwean youth 13 to 17 years of age living in households with or without their biological parents, and we assess the utility and limitations of existing data.

**Method:**

This study is a secondary analysis of nationally representative Violence Against Children Surveys (N = 6,405). Logistic regressions examined the effect of alternative care experience on the odds of poor outcomes, controlling for covariates including parental care status, orphanhood, and household assets.

**Results:**

In both bivariate and multivariate analyses, having lived in alternative care in the last 5 years was associated with lowered odds of mental distress (odds ratio [OR] = 0.25, 95% CI = [0.10, 0.61], *p* = .002), and higher odds of sexual risk taking (OR = 1.70, 95% CI = [1.11, 2.59], *p* = .014), caregiver physical abuse (OR = 1.81, 95% CI = [1.30, 2.50], *p* < .001), caregiver emotional abuse (OR = 1.75, 95% CI = [1.20, 2.54], *p* = .004), and peer violence (OR = 1.57, 95% CI = [1.09, 2.26], *p* = .015). It was not associated with suicidality, self-harm, or sexual assault after controlling for covariates.

**Conclusion:**

Youth who have lived in alternative care in the last 5 years may benefit from programs that address violence, self-harm, and sexual risk taking behavior, even if they are now in families. To better understand children outside parental care, national data collection efforts should distinguish between residential and family-based care.

In sub-Saharan Africa, the diversity of child living arrangements often challenges Western assumptions about family structure and caregiving.^1^ African family structures can range from a small nuclear family to mixed families with multiple parents and children or the co-habitation of extended families within the same space.^2^^,^^3^ Care arrangements are also diverse when children’s biological parents are unable to care for them, with kin and communities often filling this role.^4^ Recent years have seen increases of children living apart from their parents (often termed “alternative care”) because of factors such as poverty, migration, conflict, natural disasters, and health crises.^5^, ^6^, ^7^, ^8^ For example, countries with higher adult HIV prevalence have higher orphanhood rates, and the risk of orphanhood increases by 6% in areas with armed conflict.^9^^,^^10^

Alternative care consists of formal (ie, ordered by a governmental authority) or informal (ie, a private arrangement) care arrangements for children whose biological parents are unable care for them or have “abandoned or relinquished” them.^8^ It can consist of kinship care, whereby a child is cared for by an extended family; foster care, a formal arrangement whereby children are cared for by a non-relative family; and residential care, whereby children live in group settings such as orphanages (also called institutions or children’s homes), group homes, or shelters.^8^ Traditionally, informal kinship care was the dominant form of alternative care in sub-Saharan Africa, but as economic stress, conflict, and health crises weakened these networks, residential care expanded.^11^^,^^12^ Importantly, many children in alternative care have living parents, and enter for socioeconomic reasons or because of family violence.^13^^,^^14^ Globally, three-fourths of children who live in households without their parents actually have 2 living parents.^13^ Some of these children live in child-headed households with no adults present, although they often have living parents as well.^13^

The present study aims to examine violence, sexual risk taking, and mental health outcomes for children who have experienced alternative care in 3 sub-Saharan African nations: namely, Nigeria, Zambia, Zimbabwe. Each of these countries has unique factors that affect alternative care for children within their borders, but they also share similarities.

### Alternative Care in Nigeria, Zambia, and Zimbabwe

Nigeria, Zambia, and Zimbabwe each based their alternative care policies on the United Nations Convention on the Rights of the Child, which pronounces that children should live in family settings rather than institutions whenever possible.^15^, ^16^, ^17^, ^18^, ^19^, ^20^, ^21^ Similar phenomena drive children into alternative care in all 3 countries, including poverty, family breakdown, child maltreatment, sociopolitical instability, and HIV/AIDS.^17^^,^^22^, ^23^, ^24^, ^25^, ^26^ Children in alternative care are usually in kinship care, with relatively fewer children in institutions.^17^^,^^25^ All 3 countries were formerly colonized by the British; 1 researcher posits that Zimabwean alternative care practices were strongly influenced by colonial rule, with early residential institutions established according to Christian religious values of caring for disadvantaged individuals, and this could plausibly be true of Zambia and Nigeria as well.^27^ Indeed, institutions in all 3 countries may be run by non-governmental organizations, including churches.^22^^,^^27^ However, there are also differences across the 3 countries. Zimbabwe has the highest prevalence of children in alternative care, with 24% of children living with neither biological parent and 113 per 1,000 children in institutions^28^; similarly, in Zambia, 17% of children are in nonparental care and 100 per 1,000 in institutions^25^^,^^28^; in Nigeria the numbers are much lower, with 10% of children in nonparental care and only 4 per 1,000 in institutions.^28^^,^^29^ In addition, the issues of illegally operating institutions and “baby factories” are uniquely prominent in Nigeria,^22^ whereas rates of HIV/AIDS are much higher in Zambia and Zimbabwe than in Nigeria.

### Child Outcomes Research and Alternative Care

Like elsewhere in the world, children and adolescents 13 to 17 years of age in sub-Saharan Africa may experience psychological distress due to stressors around puberty, the transition to adulthood, and the pressures of educational achievement. There is also rising concern about the mental health of children in sub-Saharan Africa in particular, because of increasing economic hardship, climate change, conflicts, displacement, and under-investment in mental health supports.^30^ A growing body of literature also examines the mental health, violence, and sexual risk taking outcomes of children who have lived in alternative care across the subcontinent. Some studies have compared children who live with their parents to those in families without their parents. In Rwanda, after controlling for HIV status, children living in foster care (mostly kinship care) had more symptoms of depression, anxiety, and irritability than children living with their parents.^31^ In a study of 5 African nations, children who did not live with a parent were also found to be more likely to engage in risky sexual behavior than those who did.^32^ Another study, which included 3 African and 2 non-African countries, found that 84% of children residing apart from their living parents had experienced sexual abuse, a rate higher than that of the orphaned and non-orphaned children in the study.^33^ These outcomes may occur because kinship caregivers are stretched thin by having to care for relatives’ children, and thus children receive less individualized care and supervision.^32^ Prior research has also found that adults who care for orphaned children experience higher rates of depression than caregivers whose children were not orphans, which could also affect caregiving quality.^34^ We could not, however, identify studies of children who had left kinship or foster care and returned to parents.

Other studies have examined outcomes for children in residential care. One study of residential facilities for street-connected children in Zambia found that three-fourths had a mental health problem, potentially due to high rates of risk factors such as poverty, interrupted attachment relationships, and experiences of maltreatment.^35^ Young people in Zimbabwe have reported that they are stigmatized as “orphans” due to living in residential care, and that internalizing this label reduces their self-esteem.^36^ According to other interviews in Zimbabwe, caregivers and practitioners believe that foster care provides children with better socialization opportunities than institutional care, leading to better adjustment.^37^ Looking at the subcontinent more broadly, in Kenya, children in alternative family-based care had greater internalizing problems than those in residential care.^38^^,^^39^ This research also found that children in residential care were less likely to engage in transactional sex or to experience sexual abuse than those in family care.^40^^,^^41^ In Rwanda, children in residential care also had lower mental distress and risk-taking behavior than those in foster families.^42^ Studies in various African countries have found that when looking at similarly vulnerable children, those in residential care tended to have greater access to material resources, such as education, nutrition, and shelter, than those in families.^43^, ^44^, ^45^ This also holds true for children who first lived in residential care but then reunified with family.^44^, ^45^, ^46^ A study in Ghana also revealed that despite having fewer material resources, reunified children had more hope than those in residential care, possibly because closeness with family is protective.^46^ Taken together, although children in residential care likely have worsened mental health compared to that of the general population because of pre-existing risk factors or harmful experiences in residential care, they may experience a higher quality of care than children with foster families or relatives.

Overall, the literature suggests that children currently or formerly in all types of alternative care in sub-Saharan Africa have heightened risk of adversity, but a great deal of uncertainty remains, and outcomes can differ by care type. Large-scale data collection efforts by governments could potentially shed light on these questions.

### Systematic Data Collection on Alternative Care in Africa

Large-scale data on children who have lived in alternative care in sub-Saharan Africa remain relatively scarce. Currently, the most prominent data sources are the UNICEF Multiple Indicator Cluster Surveys and the USAID Demographic and Health Surveys.^13^ These repeated cross-sectional household surveys are employed in nearly all regions of the world, using clustering sampling designs that allows them to collect nationally representative data. Their aims are broad, examining populational-level trends in health, education, and economic status; unfortunately, their structure does not allow for a clear understanding of the types of relationships between children and adults in a household or the current and prior alternative care status of children.^13^ The surveys also exclude children living in residential care, as they sample only households.

More recently, UNICEF, with USAID support, developed the *Protocol for a National Census and Survey on Children in Residential Care* for collecting rigorously sampled and internationally standardized data on a country’s residential care institutions and the children living in them.^14^ The protocol, which has been piloted in 3 countries, is a crucial contribution to understanding the landscape of alternative care for children; however, it examines only the current situation of children in residential care facilities, and does not include children in family-based alternative care or children who have left residential care and joined families.

In 2007, the Together for Girls and the Centers for Disease Control and Prevention began conducting nationally representative household surveys called the Violence Against Children Surveys (VACS).^47^ More narrow in scope than other household surveys but broader than the residential care initiative, the VACS collects detailed data about violence perpetration and risk and protective factors in regard to violence. Importantly, when the VACS were conducted in Nigeria and Zambia in 2014, the questionnaires included an item for children under 18 about alternative care: whether the respondent had “lived outside of family care in the last 5 years[.] For example an orphanage, shelter or foster care, or with other relatives/families/friends.” This item also appeared in the Zimbabwe VACS in 2017*.* Thus, to our knowledge, the Nigeria, Zambia, and Zimbabwe VACS are the first, and currently the only, publicly available nationally representative datasets with information about African children who have experienced alternative care.

Therefore, the aim of this study is to explore whether having previously lived in alternative care is linked to adverse outcomes and experiences—including mental distress, self-harm, suicidality, sexual risk taking, sexual assault, caregiver physical abuse, caregiver emotional abuse, and peer violence—in a sample of Nigerian, Zambian, and Zimbabwean children living in households, after controlling for demographic covariates and potential confounders, such as economic status, parental death, and parental care status. We hypothesized that respondents who had lived in alternative care in the past 5 years would have poorer outcomes than those who had not. We also assessed the utility and limitations of these data as they are currently collected.

## Method

This cross-sectional study uses VACS from Nigeria and Zambia (collected in 2014), and Zimbabwe (collected in 2017), as they were the only publicly available VACS in sub-Saharan Africa that included the question about alternative care.^47^ The VACS included 13- to 24-year-old individuals using multi-stage sampling designs with random selection of a regional cluster, probability systematic sampling of households within clusters, and random selection of a child respondent from within the household. Since they are household surveys, all children were living in some type of family at the time of the survey, and none were in residential care. All child respondents and their heads of household completed the survey in their home via interview with an enumerator; in Nigeria and Zambia, these could take place over the course of up to 3 visits, but the number of visits was not recorded in Zimbabwe. Additional information about survey procedures has been published elsewhere.^47^ We excluded respondents over age 17 years of age, as the VACS presented the question about alternative care only to children and adolescents under 18 years. As nationally representative surveys stratified by region, each sample was likely ethnically diverse; Nigeria participants reported belonging to 26 distinct ethnic groups, although the Zambia and Zimbabwe surveys did not collect ethnicity data. The resulting sample included N = 6,405 observations, with n = 1,840 from Nigeria, n = 785 from Zambia, and n = 3,780 from Zimbabwe. We weighted the data so that each country was equally weighted.

### Measures

#### Dependent Variables

All dependent variables were binary. Variables calculated from multiple items were coded as missing if any of their items were missing.

Mental distress was measured using the Kessler Scale of Psychological Distress, which contains six 5-level Likert questions about frequency of internalizing symptoms (Cronbach α = .84). Mental distress was dichotomized according to US norms, coded as 1 if the total score was above 13 and as 0 if it was 12 or below.^48^

Self-harm was measured by whether respondents answered “yes” to the question “Have you ever intentionally hurt yourself in any way?” Suicidality was whether respondents reported that they had ever thought about killing themselves or had tried to kill themselves.

The sexual risk taking variable was whether the respondent reported having had 2 or more sexual partners, had ever engaged in transactional sex, or had had sex without a condom in the past 12 months. Sexual assault was whether the respondent reported that they had ever experienced rape, attempted rape, coerced or pressured someone for sex, or engaged in unwanted touching.

Caregiver physical abuse indicated whether the respondent reported that a parent, adult caregiver, or other adult relative had ever “punched, kicked, whipped, or beat [them] with an object”; choked, suffocated, tried to drown [the respondent], or burned [them] intentionally”; or “used or threatened [them] with a knife or other weapon.” The caregiver emotional abuse variable was whether the respondent reported that a parent, adult caregiver, or other adult relative had ever “told [them] that [they] were not loved, or did not deserve to be loved”; “said they wished [they] had never been born or were dead”; or “ridiculed [them] or put [them] down, for example said that [they] were stupid or useless.”

Finally, peer violence was a measure of whether the respondent reported that “a person [their] own age” had ever “punched, kicked, whipped, or beat [them] with an object”; “choked, suffocated, tried to drown [the respondent], or burned [them] intentionally”; or “used or threatened [them] with a knife or other weapon.”

### Independent Variables

The VACS asked if a child had experienced alternative care by asking their head of household “Has [the child] lived outside of family care in the last 5 years? For example an orphanage, shelter or foster care, or with other relatives/families/friends?” Thus, this variable was binary.

Parental care status indicated whether the child currently lived with both biological parents (reference group), their biological mother only (maternal care), biological father only (paternal care), or neither biological parent (non-parental care). Orphanhood was whether the child’s parents were both deceased (double orphan), mother only was deceased (maternal orphan), father only was deceased (paternal orphan), or both parents were alive (non-orphan; reference group).

Also included as controls were sex (female = 1, male = 0), age, highest education level completed (less than primary = 0, primary = 1, secondary = 2, higher than secondary = 3), country, and a household assets score. Assets score served as a proxy of respondents’ economic status and was a sum of the number of the following belongings that the head-of-household reported possessing: electricity, radio, television, telephone, refrigerator, watch, motorcycle/scooter, or car/truck (α = .74, range, 0-8). (These assets were used because they were present across all 3 questionnaires. Three items were excluded due to reducing scale internal consistency: paraffin lamp, bicycle, and oxcart.)

### Data Analysis

We analyzed data in Stata 17 SE.^49^ The VACS use complex sample designs with clustering, stratification, and sample weights, which were applied using the *svy* command and *subpop* option to all analyses except calculations of the Cronbach alpha.

To examine the associations between alternative care experience and outcomes, we conducted cross-tabulations and evaluated the design-based Rao−Scott correction of the Pearson χ^2^ statistic. We then conducted 8 binary logistic regressions with the predictors alternative care experience, parental care status, orphanhood, sex, household assets, age, and education level, to see whether associations remained. As a sensitivity analysis, we conducted the same logistic regressions but systematically checked for interactions between alternative care experience and the 6 control variables to determine whether there were any crossover interactions affecting the statistical significance of alternative care experience. Missing cases were handled with listwise deletion, and model multicollinearity was assessed by ensuring that variance inflation factors were below 10.

## Results

Descriptive statistics for the samples are presented in Table 1. Overall, 8% of children had alternative care experience: 7% of children in Nigeria, 11% in Zambia, and 6% in Zimbabwe. About 22% of the sample lived with neither parent, and 5% were double orphans. Approximately 4% met the criteria for mental distress, and as many as 31% had experienced caregiver physical abuse. Children’s parental care status varied significantly by alternative care status (*F*_2.49,3719.21_ = 14.55, *p* < .001); alternative care−experienced children lived with neither of their parents at twice the rate of other children (Table 2). Orphanhood status also varied significantly by alternative care experience (*F*_2.92,4350.69_ = 4.94, *p* = .002), with children who had lived in alternative care being more likely to be orphaned than other children (Table 3).

**Table 1:** Descriptive Statistics, by Country and Overall

	Nigeria	Zambia	Zimbabwe	Overall	% Missing
Alternative care experienced, %	7.1	11.4	6.1	8.2	1.6
Parental care status, %					0.0
Neither parent	15.2	20.6	29.8	21.9	
Maternal care	9.7	19.8	19.9	16.6	
Paternal care	5.0	5.4	7.3	5.9	
Both parents	70.1	54.2	43.0	55.6	
Orphan status, %					0.6
Non-orphan	86.0	75.7	69.5	77.0	
Paternal orphan	8.2	14.3	17.8	13.5	
Maternal orphan	4.4	5.1	5.0	4.8	
Double orphan	1.5	4.9	7.7	4.7	
Female, %	48.2	51.1	51.1	50.2	0.0
Age, mean (SD)	14.8 (1.4)	14.9 (0.8)	15.0 (1.8)	14.9 (1.3)	0.0
Household assets, mean (SD) [range, 0-8]	4.1 (1.9)	2.7 (1.2)	4.2 (1.9)	3.1 (2.1)	1.5
Education level, %					0.4
Less than primary	19.3	5.6	1.1	8.5	
Primary	21.1	58.4	30.9	37.2	
Secondary	57.9	34.4	67.6	53.1	
Higher than secondary	1.7	1.6	0.4	1.2	
Mental distress, %	3.9	7.6	1.8	4.4	4.4
Suicidality, %	3.9	7.3	4.5	5.3	0.4
Self-harm, %	6.0	9.5	1.7	5.8	0.6
Sexual risk-taking, %	14.8	20.4	65.8	19.4	3.0
Sexual assault, %	20.4	18.4	3.8	14.1	2.0
Caregiver physical abuse, %	43.5	36.0	15.1	31.3	1.2
Caregiver emotional abuse, %	25.0	27.4	11.7	21.3	0.6
Peer violence, %	32.6	20.0	14.2	22.1	1.0

**Table 2:** Respondents’ Parental Care Status, by Alternative Care Experience (Crosstabs and Chi-Squared Analyses)

	Lives with both parents	Lives with mother	Lives with father	Lives with neither parent	Total
Alternative care experienced	31.3	17.5	11.1	40.2	100
No alternative care experience	57.7	16.5	5.4	20.4	100

Note: Data are percentages. F_2.49,3719.21_ = 14.55, *p* < .001.

**Table 3:** Respondents’ Orphan Status, by Alternative Care Experience (Crosstabs and Chi-Squared Analyses)

	Non-orphan	Father deceased	Mother deceased	Both parents deceased	Total
Alternative care experienced	69.2	12.9	8.8	9.1	100
No alternative care experience	77.8	13.5	4.5	4.3	100

Note: Data are percentages. F_2.92,4350.69_ = 4.94, *p* = .002.

Bivariate associations between alternative care experience and outcome variables are presented in Table 4. Children with who had lived in alternative care in the last 5 years were less likely to have mental distress (*F*_1,1599_ = 6.36, *p* = .012) but were more likely to have engaged in self harm (*F*_1,1599_ = 5.66, *p* = .018) and sexual risk taking (*F*_1,1599_ = 16.30, *p* < .001), and to have experienced sexual assault (*F*_1,1599_ = 12.51, *p* < .001), physical abuse by a caregiver (*F*_1,1599_ = 14.57, *p* < .001), emotional abuse by a caregiver (*F*_1,1599_ = 13.31, *p* < .001), and peer violence (*F*_1,1599_ = 7.19, *p* = .007). There was no significant difference in suicidality between the 2 groups (*F*_1,1599_ = 2.96, *p* = .151).

**Table 4:** Proportion of Respondents With Mental Health Problems, Sexual Risk Taking, and Experiences of Violence, by Alternative Care Experience (Crosstabs and Chi-Squared Analyses)

	No alternative care experience	Alternative care experienced
Mental distress∗	4.7	1.7
Suicidality	5.1	7.5
Self-harm∗	5.4	9.8
Sexual risk-taking∗∗∗	18.1	32.4
Sexual assault∗∗	13.3	22.2
Caregiver physical abuse∗∗∗	30.2	43.5
Caregiver emotional abuse∗∗∗	20.2	34.4
Peer violence∗∗	21.2	29.8

Note: Data are percentages.

∗*p* < .05; ∗∗*p* < .01; ∗∗∗*p* < .001.

Multivariate analyses that controlled for demographic characteristics, including parental care and orphanhood status, can be found in Table 5. In these, children with alternative care experience still had significantly lowered odds of having mental distress (odds ratio [OR] = 0.25, 95% CI = [0.10, 0.61], *p* = .002). They also still had higher odds of engaging in sexual risk-taking behavior (OR = 1.70, 95% CI = [1.11, 2.59], *p* = .014), and of experiencing caregiver physical abuse (OR = 1.81, 95% CI = [1.30, 2.50], *p* < .001), emotional abuse (OR = 1.75, 95% CI = [1.20, 2.54], *p* = .004), and peer violence (OR = 1.57, 95% CI = [1.09, 2.26], *p* = .015). As in bivariate analyses, children did not differ on suicidality by alternative care experience (OR = 1.29, 95% CI = [0.70, 2.37], *p* = .409). Although they had in bivariate analyses, children with and without recent alternative care experience did not differ in odds of self-harming (OR = 1.64, 95% CI = [0.92, 2.92], *p* = .093) or having been sexually assaulted (OR = 1.58, 95% CI = [0.91, 0.2.74], *p* = .104) in multivariate analyses.

**Table 5:** Binary Logistic Regressions Examining Odds of Mental Health Problems, Sexual Risk Taking, and Experiences of Violence Multivariate Analyses (OR [95% CI])

	Mental distress	Suicidality	Self-harm	Sexual risk-taking	Sexual assault	Caregiver physical abuse	Caregiver emotional abuse	Peer violence
Alternative care experienced	0.25 [0.10, 0.61]∗∗	1.29 [0.70, 2.37]	1.64 [0.92, 2.92]	1.70 [1.11, 2.59]∗	1.58 [0.91, 2.74]	1.81 [1.30, 2.50]∗∗∗	1.75 [1.20, 2.54]∗∗	1.57 [1.09, 2.26]∗
Country (ref = Nigeria)								
Zambia	1.96 [1.16, 3.31]∗	1.74 [1.01, 3.00]∗	1.63 [1.09, 2.46]∗	1.11 [0.81, 1.54]	0.82 [0.57, 1.20]	0.79 [0.61, 1.00]	1.22 [0.91, 1.64]	0.58 [0.44, 0.75]∗∗∗
Zimbabwe	0.39 [0.23, 0.67]∗∗	0.98 [0.57, 1.69]	0.27 [0.16, 0.45]∗∗∗	0.18 [0.13, 0.26]∗∗∗	0.12 [0.08, 0.17]∗∗∗	0.25 [0.20, 0.33]∗∗∗	0.41 [0.31, 0.55]∗∗∗	0.37 [0.27, 0.49]∗∗∗
Parental care status (ref = both parents)								
Non-parental care	1.45 [0.66, 3.17]	0.94 [0.54, 1.66]	0.96 [0.49, 1.91]	2.60 [1.86, 3.64]∗∗∗	1.39 [0.87, 2.23]	0.95 [0.72, 1.25]	1.37 [0.98, 1.90]	1.36 [0.99, 1.87]
Maternal care	1.40 [0.76, 2.59]	1.09 [0.60, 1.98]	1.28 [0.69, 2.37]	1.23 [0.77, 1.98]	1.53 [0.82, 2.86]	1.43 [1.00, 2.03]∗	1.54 [1.03, 2.30]∗	1.24 [0.81, 1.89]
Paternal care	1.07 [0.27, 4.19]	2.26 [0.86, 5.91]	0.84 [0.37, 1.88]	0.96 [0.55, 1.70]	0.95 [0.46, 1.97]	0.76 [0.46, 1.26]	1.01 [0.57, 1.79]	0.72 [0.37, 1.41]
Orphanhood status (ref = non-orphan)								
Paternal orphan	0.78 [0.39, 1.55]	1.50 [0.86, 2.61]	1.03 [0.52, 2.05]	1.14 [0.72, 1.79]	1.34 [0.73, 2.45]	1.00 [0.70, 1.41]	1.04 [0.70, 1.55]	0.79 [0.52, 1.19]
Maternal orphan	0.81 [0.27, 2.46]	1.12 [0.47, 2.68]	1.91 [0.84, 4.35]	0.89 [0.48, 1.66]	1.85 [0.88, 3.88]	1.29 [0.79, 2.10]	1.76 [1.04, 2.99]∗	1.71 [0.94, 3.11]
Double orphan	1.54 [0.58, 4.08]	2.64 [1.40, 4.98]∗∗	1.48 [0.57, 3.81]	1.17 [0.58, 2.33]	2.26 [1.14, 4.46]∗	0.94 [0.57, 1.56]	1.04 [0.61, 1.79]	0.68 [0.40, 1.14]
Female	1.45 [0.87, 2.42]	2.84 [1.85, 4.38]∗∗∗	0.79 [0.55, 1.14]	1.39 [1.04, 1.87]∗	4.58 [3.20, 6.55]∗∗∗	1.01 [0.82, 1.24]	0.86 [0.68, 1.10]	0.49 [0.40, 0.60]∗∗∗
Assets	1.00 [0.90, 1.12]	1.07 [0.95, 1.20]	1.07 [0.97, 1.19]	0.89 [0.82, 0.95]∗∗	0.97 [0.89, 1.06]	1.08 [1.03, 1.13]∗∗	1.12 [1.05, 1.19]∗∗	1.06 [1.01, 1.12]∗
Age	1.12 [0.98, 1.29]	1.24 [1.08, 1.43]∗∗	1.14 [0.99, 1.32]	1.97 [1.76, 2.22]∗∗∗	1.33 [1.20, 1.48]∗∗∗	0.92 [0.85, 0.98]∗	1.11 [1.03, 1.20]∗∗	0.92 [0.85, 0.99]∗
Education level	0.96 [0.71, 1.29]	1.09 [0.71, 1.67]	1.05 [0.78, 1.41]	0.61 [0.49, 0.75]∗∗∗	1.33 [1.03, 1.73]∗	1.09 [0.93, 1.27]	1.07 [0.88, 1.29]	1.15 [0.97, 1.36]
Constant	0.01 [0.00, 0.06]∗∗∗	0.00 [0.00, 0.00]∗∗∗	0.01 [0.00, 0.05]∗∗∗	0.00 [0.00, 0.00]∗∗∗	0.00 [0.00, 0.00]∗∗∗	1.54 [0.56, 4.21]	0.03 [0.01, 0.11]∗∗∗	1.16 [0.40, 3.35]
No. of observations	5,950	6,156	6,150	6,071	6,117	6,132	6,140	6,143
*F*_13,1587_	5.14∗∗∗	4.35∗∗∗	7.50∗∗∗	26.17∗∗∗	18.06∗∗∗	15.34∗∗∗	9.50∗∗∗	15.09∗∗∗

Note: Data are odds ratios [95% CIs]. ref = reference.

∗*p* < .05; ∗∗*p* < .01; ∗∗∗*p* < .001.

To further investigate the mental distress model, the result of which was contrary to our hypothesis, we ran the same analysis but stratified by country. Alternative care experience was not a significant predictor in models for Zimbabwe alone (OR = 0.69, 95% CI = [0.22, 2.19], *p* = .531) or Nigeria alone (OR = 0.24, 95% CI = [0.06, 1.02], *p* = .053), but it was in the Zambia-only model (OR = 0.23, 95% CI = [0.07, 0.78] *p* = .019).

Sensitivity analyses indicated no cross-over interaction effects, and there were no concerns with multicollinearity according to the variance inflation factors statistic.

## Discussion

This study is the first, to our knowledge, to use nationally representative data to look at youth with alternative care experience anywhere in sub-Saharan Africa. Our results indicate that individuals 13 to 17 years of age who had been in alternative care in the last 5 years were at heightened risk for experiencing violence (caregiver emotional and physical abuse as well as peer violence), as well as for sexual risk taking, but, surprisingly, were protected from mental distress. These results were seen even when controlling for demographic covariates, suggesting that having recently been in alternative care is uniquely predictive of these outcomes, rather than being confounded by orphanhood, parental care status, household economic status, or other demographic factors.

Our finding that children who have experienced alternative care are at risk for sexual assault and peer violence reinforces literature showing that children in alternative care settings experience high rates of abuse.^40^ Our finding that children who had been in alternative care had lowered odds of mental distress was contrary to our hypothesis; when we stratified by country, children who had experienced alternative care had lower odds of mental distress in all countries, but the relationship reached statistical significance only in Zambia. One Zambian study found that three-fourths of children in residential care had a mental health problem,^35^ but the prevalence of mental health problems among children in family-based alternative care is unknown. Thus, it is possible that the positive mental health outcomes in our study were driven by Zambian children in family-based, nonparental care arrangements. Looking to other published literature to explain our study findings presents difficulties, as there are few exact parallels. Prior studies found that children in residential care have greater access to resources than those who remain in vulnerable families; however, these studies combine children living with biological parents and those in nonparental family-based care, whereas our study combines children who have lived in nonparental, family-based alternative care with those who have lived in institutions.^43^^,^^44^ Data in Kenya suggest that children may escape family violence by finding shelter in residential care facilities, and show that children in residential care have better mental health than similarly vulnerable children in family settings; however, these studies combine children in nonparental family-based care with single orphans living with a biological parent.^38^, ^39^, ^40^ On the other hand, a study in Rwanda found that children in foster care had greater mental health problems than those living with their biological parents; although this contradicts our findings, our analysis combined children who have lived in foster care with those who have lived in residential care.^31^ Thus, although the significant differences that we uncovered between children who had experienced alternative care and those who did not suggest that more attention must be paid to this issue, the specific drivers behind these findings cannot be untangled.

This is because although the VACS collects detailed data on abuse perpetration, it does not indicate when a child was in alternative care, or provide enough granularity to ascertain whether abuse occurred prior to entering alternative care, while in it, or after leaving. One possibility is a child could have experienced violence or adversity in their original families, and entered alternative care as a result. They also may have experienced adversity in a foster home, kinship care, or residential institution. In addition, at the time of the survey, children may or may not have been in alternative care: although none were in residential care (as the VACS samples only households), 40% of children who were reported to have been in alternative care in the last 5 years were living apart from their parents when they were surveyed, meaning that they were still in foster or kinship care. Adverse outcomes may have occurred as a result of children’s experiences before, during, or after alternative care.

One important opportunity for increasing the usefulness of the VACS data on alternative care would be to collect separate information about family-based and residential care. Although the survey question in the VACS frames alternative care as “an orphanage, shelter or foster care, or with other relatives/families/friends,” research across the subcontinent has often found that residential care and family-based care have distinct effects on children, as we previously explored.^31^^,^^39^^,^^42^^,^^43^ Many sub-Saharan African nations are engaging in reforms and conversations about the role of residential care and family-based alternative care in services for vulnerable children, and many have set, or are in the process of developing, policies that transition away from the use of orphanages and toward the use of family-based care, including by moving children currently in residential care back into their original families or into foster families. If the survey question were split, with 1 question asking about children’s experience of residential care and 1 question asking about alternative family care, VACS data could be more useful for examining the linkages between care type and child outcomes. For example, questions could be framed as follows:

(1) *In the past 5 years, did the child live in an orphanage, shelter, or other facility? (A facility is a place where 11 or more children who are not related by blood to their caregiver are living.)*
(2) *In the past 5 years, did the child ever live temporarily with someone other than you? (That is, in foster care or with other relatives/families/friends, in a household where they did not have a biological or adoptive parent present.)*

This phrasing could rectify another issue, which is the possible multiple understandings of the question by respondents. Although heads of households were asked, “Did [child] live outside of family care in the last 5 years, for example an orphanage, shelter or foster care, or with other relatives/families/friends?” the results showed that they responded “yes” for only 16% of double orphans. It is unclear what proportion of heads of households interpreted “other relatives/families/friends” to include themselves and what proportion thought that it meant only people other than themselves.

Other study limitations include that binary outcome variables provide less precision and nuance than continuous variables would have provided. We also analyzed data from only 3 countries, and results may not be generalizable to sub-Saharan Africa’s 46 nations. Covariates that were not available to us, but that would have been useful to include, are, for example, caregiver psychosocial functioning, the length of time that the child had been living in their current living arrangement, and the frequency or timing of children moving from 1 living arrangement to another.

This study suggests that policymakers in Nigeria, Zambia, Zimbabwe, and potentially other nations, should consider children who have experienced alternative care when targeting interventions for violence, self-harm, and sexual risk. Many countries in the region are undertaking initiatives to move children out of residential care and into families,^46^ and our findings suggest that interventions to prevent violence, child maltreatment, and youth risk behaviors should be integrated into such programs. In addition, countries should prioritize the uptake of the UNICEF protocol for enumerating residential care institutions, as household surveys such as the VACS do not include children currently outside of households (eg, in residential care).^14^ Finally, future research could be designed that would specifically target young adults who grew up in alternative care, using retrospective surveys to understand the prevalence, incidence, and timing of child maltreatment during different types of alternative care.

As countries reform their alternative care systems and reunify children living in residential care with family, it is crucial to monitor outcomes for children who have experienced residential care. If the VACS are modified to distinguish between family-based and residential-based alternative care, they could be a powerful tool for providing insight into the status of children affected by such reforms. As it stands, however, these data indicate that the existing population of children who have lived in foster care, shelters, orphanages, kinship care, and other nonparental care settings should be considered when planning the provision of services for preventing violence, recovering from violence, preventing self-harm, and reducing sexual risk-taking behavior.
